# *Variovorax terrae* sp. nov. Isolated from Soil with Potential Antioxidant Activity

**DOI:** 10.4014/jmb.2205.05018

**Published:** 2022-06-30

**Authors:** Chae Yung Woo, Jaisoo Kim

**Affiliations:** Department of Life Science, College of Natural Sciences, Kyonggi University, Suwon 16227, Republic of Korea

**Keywords:** *Variovorax terrae*, *Comamonadaceae*, soil, antioxidant

## Abstract

A white-pigmented, non-motile, gram-negative, and rod-shaped bacterium, designated CYS-02^T^, was isolated from soil sampled at Suwon, Gyeonggi-do, Republic of Korea. Cells were strictly aerobic, grew optimally at 20-28ºC and hydrolyzed Tween 40. Phylogenetic analysis based on 16S rRNA gene sequence indicated that strain CYS-02^T^ formed a lineage within the family *Comamonadaceae* and clustered as members of the genus *Variovorax*. The closest members were *Variovorax guangxiensis* DSM 27352^T^ (98.6% sequence similarity), *Variovorax paradoxus* NBRC 15149^T^ (98.5%), and *Variovorax gossypii* JM-310^T^ (98.3%). The principal respiratory quinone was Q-8 and the major polar lipids contain phosphatidylethanolamine (PE), phosphatidylethanolamine (PG), and diphosphatidylglycerol (DPG). The predominant cellular fatty acids were C_16:0_, summed feature 3 (C_16:1_*ω*7c and/or C_16:1_*ω*6c) and summed feature 8 (C_18:1_*ω*7c and/or C_18:1_*ω*6c). The DNA GC content was 67.7 mol%. The ANI and dDDH values between strain CYS-02^T^ and the closest members in the genus *Variovorax* were ≤ 79.0 and 22.4%, respectively, and the AAI and POCP values between CYS-02^T^ and the other related species in the family *Comamonadaceae* were > 70% and > 50%, respectively. The genome of strain CYS-02^T^ showed a putative terpene biosynthetic cluster responsible for antioxidant activity which was supported by DPPH radical scavenging activity test. Based on genomic, phenotypic and chemotaxonomic analyses, strain CYS-02^T^ was classified into a novel species in the genus *Variovorax*, for which the name *Variovorax terrae* sp. nov., has been proposed. The type strain is CYS-02^T^ (= KACC 22656^T^ = NBRC 00115645^T^).

## Introduction

The genus *Variovorax* was reclassified in 1991 [1], and since then, some studies have demonstrated the roles of this genus in biotransformation, biosynthesis and bioremediation in ecology. For example, *Variovorax paradoxus* was reported to produce 1-aminocyclopropane-1-carboxylate deaminase, which supports plant growth [2-4]. *Variovorax boronicumulans* has also been found to produce lipopeptide siderophores (variochelin A and B) [5] that might be useful in culturing “unculturable” bacteria and is reported to synthesize indole-3-acetamide and indole-3-acetic acid, which promote plant growth using indole-3-acetonitrile as the precursor [6]. Degradation of quorum sensing signals has also been demonstrated by *Variovorax* [7, 8]. Moreover, another study reported a biosynthesis of AgNPs that have antimicrobial activity with potential application as an antibacterial agent against multidrug-resistant bacteria by *Variovorax guangxiensis* THG-SQL3 [9]. Thirteen valid *Variovorax* type strains have been described to date (http://www.bacterio.net/variovorax.html). This genus of bacteria contains summed feature 3 (C_16:1_ *ω*7c and/or C_16:1_ *ω*6c), summed feature 8 (C_18:1_ *ω*7c and/or C_18:1_ *ω*6c), C17:0 cyclo, C_16:0_, and C_10:0_ 3-OH as the major fatty acids. Polar lipid profiles of some species in the genus *Variovorax* have been shown to consist of diphosphatidylglycerol, phosphatidylglycerol, and phosphatidylethanolamine [10-13].

## Materials and Methods

### Isolation and Cultivation

The novel strain CYS-02^T^ was isolated from soil located at Suwon, Gyeonggi-do, Republic of Korea (37º15'37'' N and 126º56'17.2'' E). A modified culture method by 6-well Transwell plates (Corning, USA) was used for isolation. The Transwell plates were incubated in a shaker at 120 ×*g* for 4 weeks at 28°C. After 4 weeks, the culture was serially diluted, and 100 μl of each dilution was spread onto R2A agar plates. Colonies were individually restreaked on R2A agar plates until pure colonies were obtained. For long-term preservation, cultures were stored as suspensions in R2A broth supplemented with 20% (v/v) glycerol at –70°C. Isolation, maintenance, and preservation of strain CYS-02^T^ were performed following the methodology described by Dahal RH *et al*. [14].

### Phylogenetic Analysis

The 16S rRNA gene of strain CYS-02^T^ was extracted using the InstaGene Matrix Kit (Bio-Rad, USA) as per the manufacturer’s instructions. Isolation of 16S rRNA gene was determined by PCR using primers 27F and 1492R [15]. Sequencing was carried out by using an Applied Biosystems 3770XL DNA Analyser with a BigDye Terminator cycle sequencing Kit v.3.1 (Applied Biosystems, USA). Near-complete sequences of 16S rRNA genes (1457 bp) were assembled with SeqMan software (DNASTAR Inc.). The closest phylogenetic neighbors were identified using the EzBioCloud database [16]. All the 16S rRNA gene sequences of the closest phylogenetic members were retrieved from the NCBI GenBank database and aligned using SILVA alignment [17]. Phylogenetic trees were reconstructed using MEGA X software [18].

### Morphological, Physiological and Biochemical Analysis

The morphology of strain CYS-02^T^, grown on R2A agar for 3–4 days at 28°C, was studied by transmission electron microscopy (Talos L120C; FEI, USA). Colony morphology of all the strains was examined using a Zoom Stereo Microscope (SZ61; Olympus, Japan). Gram staining was performed according to the procedure described by Doetsch RN [19]. Motility was observed using sulfide indole motility medium (SIM; Oxoid, United Kingdom). Catalase activity was determined in 3% hydrogen peroxide. Oxidase activity was observed using 1% tetra-methyl-*p*-phenylenediamine dihydrochloride. Growth at various temperatures (4, 10, 15, 20, 28, 30°C) on R2A agar plates was monitored for 7 days. Growth was examined on various media including R2A agar (MB Cell; KisanBio, South Korea), nutrient agar (NA; Oxoid), tryptone soya agar (TSA; Oxoid), sorbitol MacConkey agar (MA; Oxoid), and Luria-Bertani agar (LBA; Oxoid). Salt tolerance was determined in R2A broth supplemented with NaCl (0–7%, at 1% interval). The pH range for growth was observed at 28°C in R2A broth adjusted to pH 4–10 (in increments of 0.5 pH units) using citrate/NaH_2_PO_4_ buffer (for pH 4.0–5.5), phosphate buffer (for pH 6–7.5) and Tris buffer (for pH 8–10) [20]. Hydrolysis of Tween 40 and Tween 80 was done using the method of Smibert & Krieg [21]. Anaerobic growth was assessed on R2A agar at 28°C for 10 days using the BD GasPak EZ Gas Generating Pouch System. A DNase activity assay was tested with DNase agar (Oxoid). Other physiological and biochemical analyses were determined using API 20NE test kits (bioMérieux, France). Enzyme activities were performed using an API ZYM Kit (bioMérieux) as suggested in the manufacturer’s instructions.

### Chemotaxonomic Analysis

Fatty acids of strain CYS-02^T^ and reference strains were harvested from same culture condition (at 28°C for 4 days) during the late log phase. Fatty acids were extracted by the standard MIDI protocol (Sherlock Microbial Identification System, version 6.0B), analyzed with a gas chromatograph (HP 6890 Series GC System; Hewlett Packard, Country), and identified using the TSBA6 database of the Microbial Identification System [22]. Polar lipids and isoprenoid quinones were extracted from freeze-dried cells according to procedures described by Minnikin *et al*. [23]. Appropriate detection reagents were used to identify the spots [24].

### Genomic and Genotypic Characterization

Whole genome-based approaches were used to confirm the taxonomic status of novel strains. For whole-genome sequencing, genomic DNA was extracted using DNeasy Blood and Tissue kits (Qiagen, Germany). Whole-genome shotgun sequencing of strain CYS-02^T^ was performed by Macrogen (Republic of Korea) using the Illumina HiSeq platform and assembled by SPAdes [25]. The authenticity of the genome assembly was checked by comparing 16S rRNA gene sequence using NCBI Align Sequences Nucleotide BLAST tool [26] and the potential contamination was checked by ContEst16S algorithm [27]. After analysis, the genome sequence was annotated utilizing the NCBI Prokaryotic Genome Annotation Pipeline [28] and Rapid Annotation using Subsystem Technology (RAST) server [29]. The anti-SMASH server was utilized to identify the biosynthetic gene clusters (BGCs) for various secondary metabolites [30]. Genome-based relatedness between strain CYS-02^T^ and closely related strains was observed based on Average Nucleotide Identity (ANI) utilizing the OrthoANIu algorithm [31]. DNA-DNA hybridization (DDH) was calculated in silico by the Genome-to-Genome Distance Calculator (GGDC 2.1) utilizing the blast method [32]. The average amino acid identity (AAI) utilizing Prodigal (Hyatt, D *et al*., 2010), MMseqs2 [33] and the EzAAI [34] were compared among strain CYS-02^T^, closely related strains, and those of the other related species in the family *Comamonadaceae*. To evaluate the percentatage of conserved proteins (POCP) analysis, open reading frames (ORFs) were performed with the Prodigal tool and reciprocal Blastp was conducted as described by Qin *et al*. [35].

### Antioxidant Activities

Screening test for antioxidant activities was achieved by DPPH (2,2-diphenyl-1-picrylhydrazyl) inhibition assay, which was performed using the method described by Dahal *et al*. [36]. The inhibitor was prepared by centrifuging the well-grown bacterial culture in R2A medium at 4°C. Ascorbic acid (Vitamin C) was taken as positive control. The assay was performed in 96-well plates and incubated at 37°C for 30 min. Absorption at 516 nm was evaluated by spectrophotometer (SpectraMax 340PC384 Microplate Reader; Molecular Devices Co., USA). Each treatment was triplicated and the percentage of radical scavenging activities was calculated using following formula:

$$\text{Inhibition(\%) = }\left[\text{1-}\frac{{\text{(OD}}_{\text{exp}}{\text{-OD}}_{\text{con}}\text{)}}{{\text{(OD}}_{\text{std}}{\text{-OD}}_{\text{bln}}\text{)}}\right]\text{×100,}$$

where: ODexp, absorbance of the experimental sample; ODcon, absorbance of control; ODstd, absorbance of standard; and ODbln, absorbance of the blank.

## Results and Discussion

### Phylogenetic Analysis

**Fig. 1:**
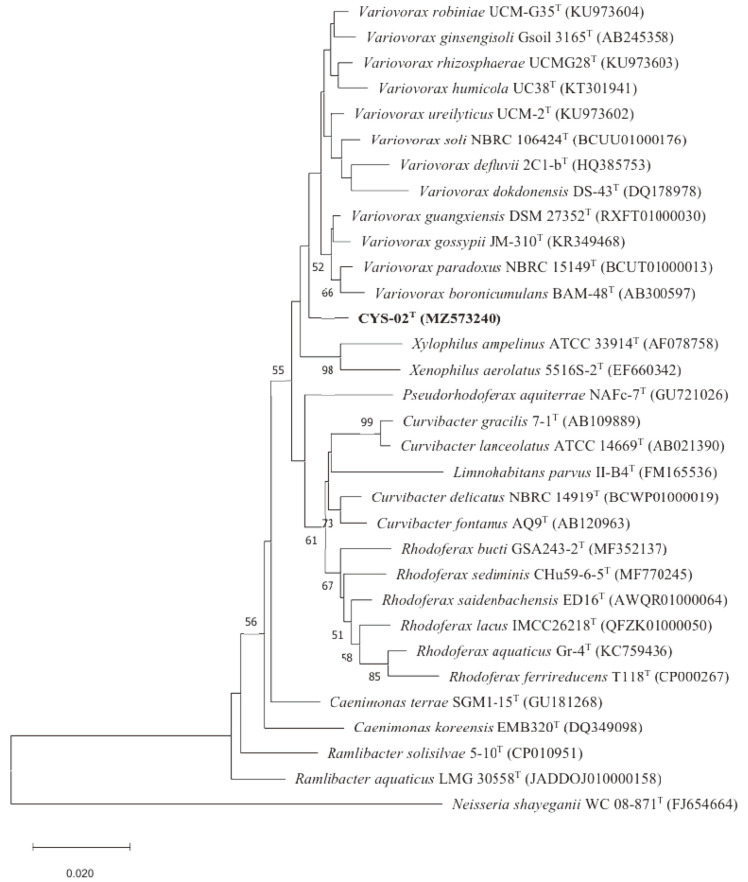
Neighbor-joining tree based on 16S rRNA gene sequences showing the phylogenetic position of strain CYS-02^T^ among closely related members of the genus *Variovorax*. The numbers at the nodes indicate the percentage of 1000 bootstrap replicates yielding this topology; only values >50% are shown. *Neisseria shayeganii* WC 08-871^T^ was used as an out-group. GenBank accession numbers are given in parentheses. Bar, 0.020 substitutions per nucleotide position.

On the basis of 16S rRNA gene sequence comparisons, strain CYS-02^T^ showed the highest similarities with *V. guangxiensis* DSM 27352^T^ (98.6% sequence similarity). Sequence similarities between strain CYS-02^T^ and other validly described *Variovorax* species were below 98.5%. Neighbor-joining (NJ), maximum-likelihood (ML) and maximum-parsimony (MP) trees with reasonable bootstrap values confirmed the identification of strain CYS-02^T^ as a novel species in the genus *Variovorax*. Furthermore, strain CYS-02^T^ was well clustered within the members of the genus *Variovorax* and formed a separate lineage with the other closest members (Figs. 1, S1 and S2).

### Morphological, Physiological and Biochemical Analysis

The colonies of strain CYS-02^T^ were rod-shaped (Fig. S3), gram-negative, strictly aerobic, non-motile, and non-sporulating. Strain CYS-02^T^ hydrolyzed Tween 80 but was unable to hydrolyze Tween 40 and DNA. Enzyme activity of acid phosphatase was positive for CYS-02^T^ but negative for reference strains (Table 1). Assimilation of D-maltose was positive for strain CYS-02^T^ but negative for reference strains (Table 1). Similarly, D-mannitol, potassium gluconate and malic acid were negative for strain CYS-02^T^ while positive for the references (Table 1). Additional physiological and biochemical differential characteristics are presented in Table 1 along with the closest members of the genus *Variovorax*.

### Chemotaxonomic Analysis

The major fatty acids of strain CYS-02^T^ were C_16:0_, summed feature 3 (C_16:1_*ω*7c and/or C_16:1_*ω*6c) and summed feature 8 (C_18:1_*ω*7c and/or C_18:1_*ω*6c), showing a similar fatty acid profile reported for the genus *Variovorax* (Table 2). The predominant respiratory quinone was ubiquinone-8 (Q-8), as it was for the reference strains. The polar lipids of the novel strain consisted of phosphatidylethanolamine (PE), phosphatidylglycerol (PG), diphosphatidylglycerol (DPG), unidentified aminophospholipids (APL), and unidentified phospholipids (PLs), including unidentified lipids (Ls) that were not reported from the reference strain *V. guangxiensis* DSM 27352^T^ [10] (Fig. S4).

### Genomic and Genotypic Characterization

The DNA GC content of strain CYS-02^T^ was 67.7 mol%, falling within the range for *Variovorax* species. The ANI threshold for species demarcation is recommended at 95–96% [37], and the Average Nucleotide Identity (ANI) values between strain CYS-02^T^ and its phylogenetically closest neighbors available with full genome sequences were ≤ 79.0% (Table S1). The dDDH values of ≤ 22.4% were much lower than the 70% species threshold recommended for species delineation [32] (Table S1), but the values for AAI and POCP were higher than 70% and 50%, respectively, indicating it to be located within the boundary of a genus [35, 38] (Table S2).

The genome of strain CYS-02^T^ is 4,934,485 bp and consists of eight scaffolds with genome coverage of 157.0× (Table S3). The RAST analysis showed the presence of 308 subsystems and 4 secondary metabolisms consisting of four plant hormones (Fig. S5). The different genomic features of the novel isolate and phylogenetically closet members of the genus *Variovorax* based on the RAST result are listed in Table S3. The general genome features are given in Table S4. The anti-SMASH analyses of BGCs observed strain CYS-02^T^ as containing 3 putative BGCs responsible for secondary metabolites such as betalactone, terpene and hserlactone (Table S5).

### Antioxidant Activities

Screening for DPPH radical scavenging activity showed the culture supernatant of strain CYS-02^T^ possessed antioxidant property. DPPH radical scavenging activity for strain CYS-02^T^ was 44% (Fig. S6). The annotation and analysis of secondary metabolite biosynthesis genes by using anti­SMASH [30] revealed that strain CYS-02^T^ contained terpene (Tables S4). This gene cluster could play important roles in antioxidant activity and its compound(s) can be used for cosmetical, pharmacological, and possible therapeutic purposes [39]. However, further investigation is needed to depict the exact bioactive chemical and pathway for antioxidant activity.

In this study, *Variovorax terrae* CYS-02^T^ was similar in major fatty acid composition, predominant ubiquinone, GC content range, and morphological analysis with other *Variovorax* type strains closely related to CYS-02^T^. Although it was distinguished from its phylogenetic neighbors due to strain CYS-02^T^ having differences based on the phylogenetic tree, polar lipids, biochemical, physiological features, and low ANI and dDDH values, it could belong to the same genus according to AAI and POCP values. In conclusion, based on genomic, chemotaxonomic, phenotypic and phylogenetic analyses, strain CYS-02^T^ represents a novel species in the genus *Variovorax*, and the name *Variovorax terrae* sp. nov. is proposed.

### Description of *Variovorax terrae* sp. nov.

*Variovorax terrae* sp. nov. (ter'rae. L. gen. n. terrae of soil, referring to the isolation source of the type strain).

Cells (1.0–2.0 μm long and 0.3–0.5 μm wide) are aerobic, gram-negative, rod-shaped, and non-motile. Cells grow well on R2A agar, LBA, NA, and TSA, and no growth is observed on MA. Colonies on R2A are white, entire, convex and circular. Colony size is 0.5–1 mm on R2A agar for 5 days at 28°C. Cells grow at 4–30°C (optimum, 20-28°C) and pH 6.0–8.0 (optimum pH, 6.5–7.0). Cells grow optimally in the absence of NaCl but tolerate 2% NaCl. Catalase and oxidase are positive. *β*-Galactosidase activity (PNPG) is negative. Esculin and Tween 40 are hydrolyzed; but DNA, urea, gelatin and Tween 80 are not. Nitrate is not reduced and glucose is not fermented. Strain SYS-02T shows the following enzyme activities: positive for alkaline phosphatase, esterase, esterase lipase, leucine arylamidase, acid phosphatase and napthol-AS-BI-phosphohydrolase. The predominant respiratory quinone is Q-8. The major cellular fatty acids are C_16:0_, summed feature 3 (C_16:1_*ω*7c and/or C_16:1_*ω*6c) and summed feature 8 (C_18:1_*ω*7c and/or C_18:1_*ω*6c). The major polar lipids contain phosphatidylethanolamine (PE), phosphatidylethanolamine (PG) and diphosphatidylglycerol (DPG). The DNA GC content calculated from the whole genome sequence of the type strain is 67.7 mol%.

The type strain is CYS-02^T^ (= KACC 22656^T^= NBRC 00115645^T^), isolated from mountain soil of Suwon in South Korea. The GenBank/EMBL/DDBJ accession numbers for the 16S rRNA gene sequence and the whole genome sequence of strain CYS-02^T^ are MZ573240 and JALGBI000000000, respectively.

## Supplemental Materials

Supplementary data for this paper are available on-line only at http://jmb.or.kr.

## Figures and Tables

**Table 1:** Phenotypic characteristics of strain CYS-02^T^ and closely related type strains of the genus *Variovorax*.

Characteristic	1	2	3	4
Maximum growth temperature (°C)	30	35^a^	40^a^	37^b^
Salt tolerance at 1% (w/v)	+	–^a^	+^a^	+^b^
pH range	6.0–8.0	5.0-9.0^a^	6.0-9.0^a^	4.5-9.0^b^
Hydrolysis of				
Tween 40	+	+^a^	–^a^	w^b^
Tween 80	–	–^a^	+^a^	–^b^
Enzyme activity				
Lipase (C14)	–	–	–	w
Valine arylamidase	–	w	–	+
Cystine arylamidase	–	–	–	w
Acid phosphatase	w	–	–	–
α-Glucosidase	–	–	+	w
Assimilation of:				
NO_2_ reduction	–	–	+	–
D-Glucose	–	+	–	w
l-Arabinose	–	+	–	w
D-Mannose	w	–	–	w
D-Mannitol	–	+	+	+
*N*-Acetyl-glucosamine	–	+	–	+
D-Maltose	+	–	–	–
Potassium gluconate	–	+	+	+
Capric acid	–	w	–	w
Adipic acid	–	–	w	–
Malic acid	–	+	w	+
DNA G + C content (mol%)*	67.7	67.4	67.7	67.4

Taxa: 1, CYS-02^T^ (this study); 2, *V. guangxiensis* DSM 27352^T^ [10]; 3, *V. paradoxus* KACC 11555^T^ [1]; 4, *V. gossypii* DSM 100435^T^ [11]. +, Positive; w, weak; –, negative.

^a^The data were obtained from the previous work [12].

^b^The data were obtained from the previous work [13].

*The DNA GC contents were calculated from the whole genome sequences in this study.

**Table 2:** Cellular fatty acid profiles (percentage of totals) of strain CYS-02^T^, closely related type strain and type species of the *Variovorax*.

Fatty acid	1	2	3	4
Saturated				
C_12:0_	3.5	3.5	3.7	4.9
C_13:0_	–	–	tr	–
C_14:0_	–	0.8	0.7	0.9
C_16:0_	33.0	25.0	27.4	31.4
C_17:0_	0.8	tr	1.1	–
C_18:0_	1.1	1.08	tr	0.9
C_20:0_	–	tr	–	–
Unsaturated				
C_15:1_ *ω*6c	–	–	1.3	–
C_16:1_ *ω*5c	–	–	0.6	–
C_17:0_ cyclo	5.7	6.6	17.2	30.8
C_19:0_ cyclo *ω*8c	–	4.1	tr	3.1
Hydroxy				
C_8:0_ 3-OH	–	0.7	0.6	–
C_9:0_ 3-OH	–	–	tr	–
C_10:0_ 3-OH	–	11.9	3.8	–
C_12:0_ 2-OH	–	tr	tr	–
C_14:0_ 2-OH	–	2.9	3.1	4.2
C_15:0_ 2-OH	–	–	tr	–
C_16:0_ 2-OH	–	tr	–	2.1
C_16:0_ 3-OH	–	–	tr	–
C_16:1_ 2-OH	–	2.4	1.5	2.5
C_18:1_ 2-OH	–	0.7	–	–
Branched-chain			
iso-C_19:0_	–	–	tr	0.6
Summed features*				
3	40.0	26.6	26.1	5.3
8	16.0	16.7	11.5	13.3

Taxa: 1, CYS-02^T^; 2, *V. guangxiensis* DSM 27352^T^; 3, *V. paradoxus* KACC 11555^T^; 4, *V. gossypii* DSM 100435^T^. All data were obtained from this study. TR, trace amount (< 0.5%); –, not detected.

*Summed features represent groups of two or three fatty acids that could not be separated using the MIDI system. Summed feature 3 comprised C_16:1_*ω*7c and/or C_16:1_*ω*6c and summed feature 8 comprised C_18:1_*ω*7c and/or C_18:1_*ω*6c.
