# Corrigendum to: *Variovorax* terrae sp. nov. Isolated from Soil with Potential Antioxidant Activity

**DOI:** 10.4014/jmb.2022.3209.1217

**Published:** 2022-09-28

**Authors:** Chae Yung Woo, Jaisoo Kim

**Affiliations:** Department of Life Science, College of Natural Sciences, Kyonggi University, Suwon 16227, Republic of Korea

In the article titled “*Variovorax terrae* sp. nov. Isolated from Soil with Potential Antioxidant Activity, the authors noticed the deposit number of NBRC was wrong, and thus are changing NBRC 00115645 to NBRC 115645 in two places within the paper. One is in the abstract (the last line; page 855) and the other is in the Description of *Variovorax terrae* sp. nov. (the first line of page 860). The corrected file is now available online.

The authors sincerely apologize for the confusion caused by this error and state that this does not change the scientific conclusions of the article in any way.

