# Supplementary material for: Variovorax terrae sp. nov. Isolated from Soil with Potential Antioxidant Activity
Source: J Microbiol Biotechnol. 2022 Jun 30;32(7):855–61. doi: 10.4014/jmb.2205.05018 (PMC9628916; doi:10.4014/jmb.2205.05018)
Supplement: Supplementary file 1 [file jmb-32-7-855-supple.pdf]

J. Microbiol. Biotechnol.
https://doi.org/10.4014/jmb.2205.05018

Supplementary Materials

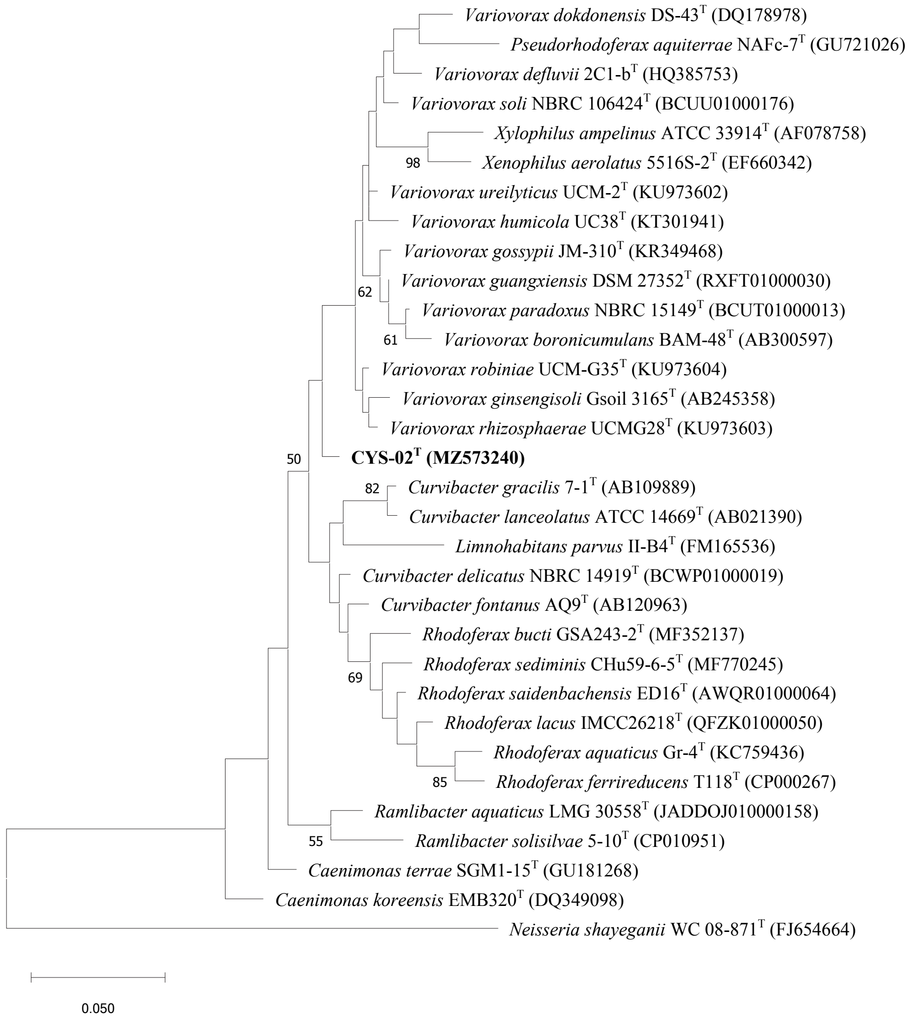

Supplementary Fig. S1. Maximum-likelihood tree based on 16S rRNA gene sequences showing the phylogenetic position strain CYS-02^T among closely related members of the genus *Variovorax*. The numbers at the nodes indicate the percentage of 1000 bootstrap replicates yielding this topology; only values >50% are shown. *Neisseria shayeganii* WC 08-871^T was used as an out-group. GenBank accession numbers are given in parentheses. Bar, 0.050 substitutions per nucleotide position.

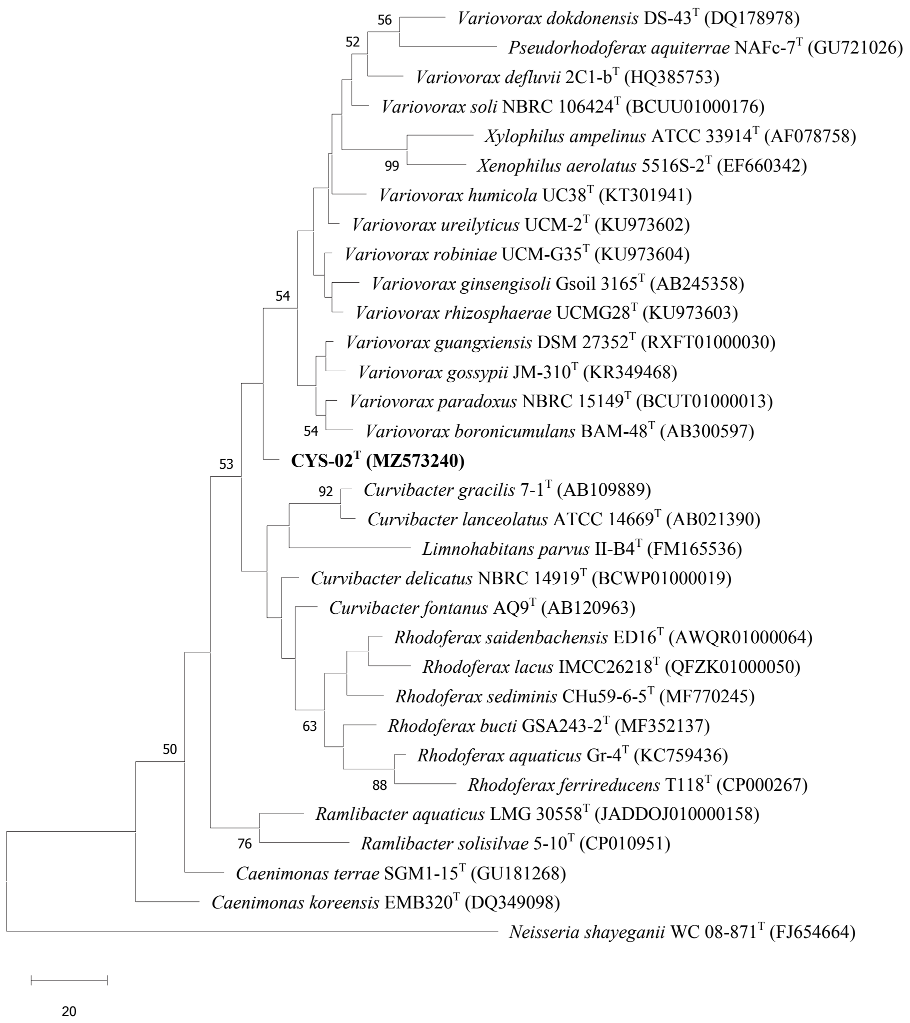

Supplementary Fig. S2. Maximum-parsimony tree based on 16S rRNA gene sequences showing the phylogenetic position strain CYS-02^{T} among closely related members of the genus *Variovorax*. The numbers at the nodes indicate the percentage of 1000 bootstrap replicates yielding this topology; only values >50% are shown. *Neisseria shayeganii* WC 08-871^{T} was used as an out-group. GenBank accession numbers are given in parentheses. Bar, 20 substitutions per nucleotide position.

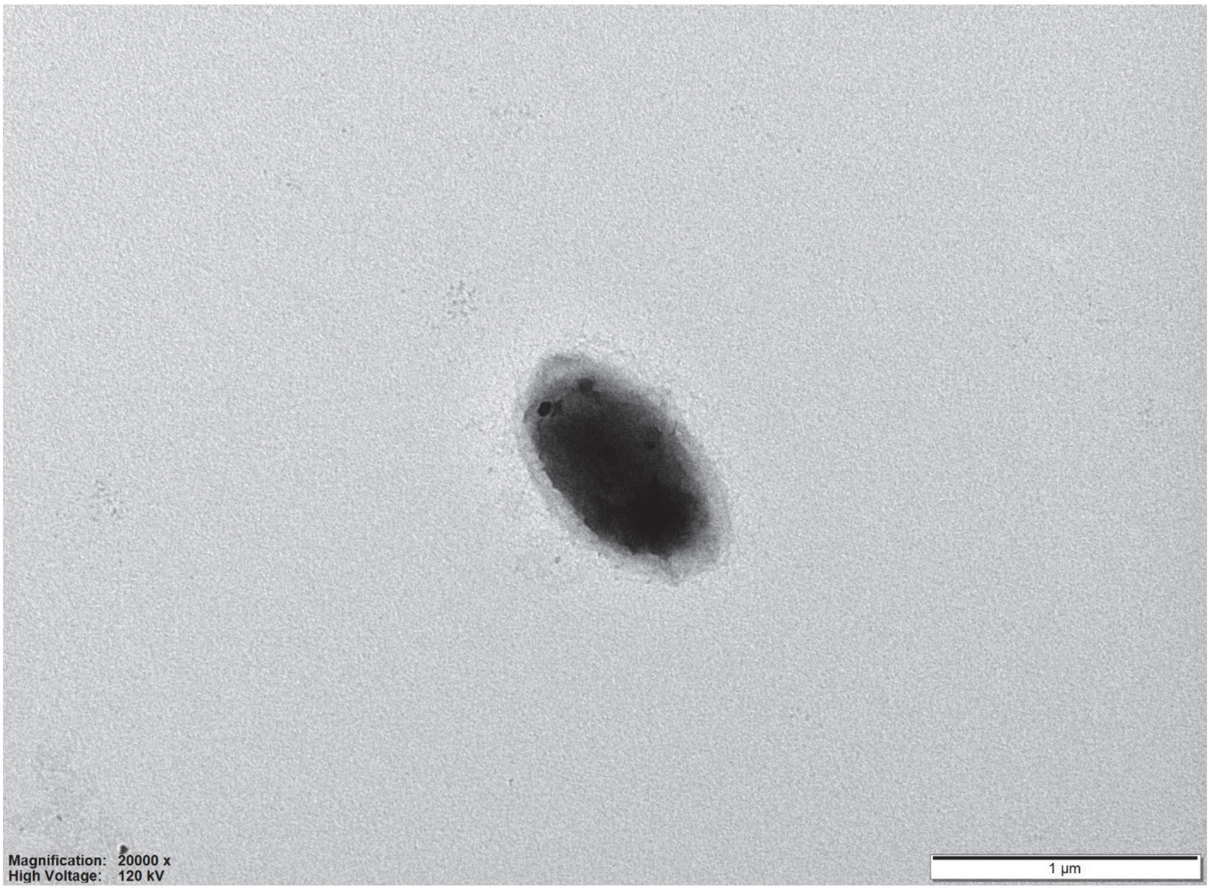

Supplementary Fig. S3. Trasmission electron photomicrograph of strain CYS-02^{T} grown on R2A agar at 28 °C for 3 days. Bar, 1 µm.

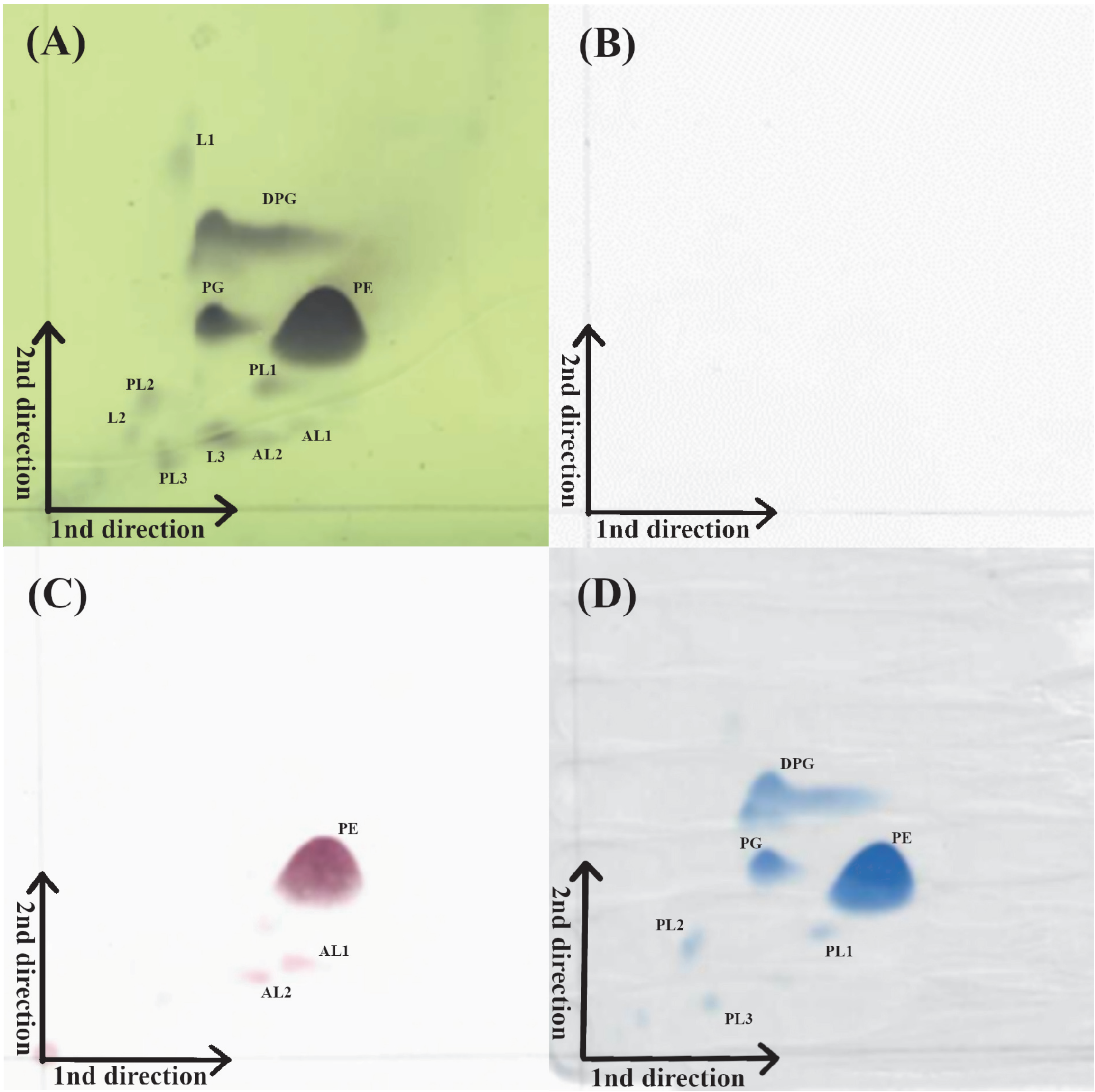

Supplementary Fig. S4. Thin-layer chromatograms of the polar lipids from strain CYS-02^T. (A) Total lipids by spraying with 5% molybdophosphoric acid in ethanol, (B) Glycolipids were revealed by α-naphthol-sulphuric acid, (C) Aminolipids detected by spraying with 0.2% ninhydrin, (D) Phospholipids detected by spraying with molybdenum blue. Abbreviations: PE, phosphatidylethanolamine; DPG, diphosphatidylglycerol; PG, phosphatidylglycerol; L, unidentified lipid; PL, unidentified phospholipid; AL, unidentified aminolipids.

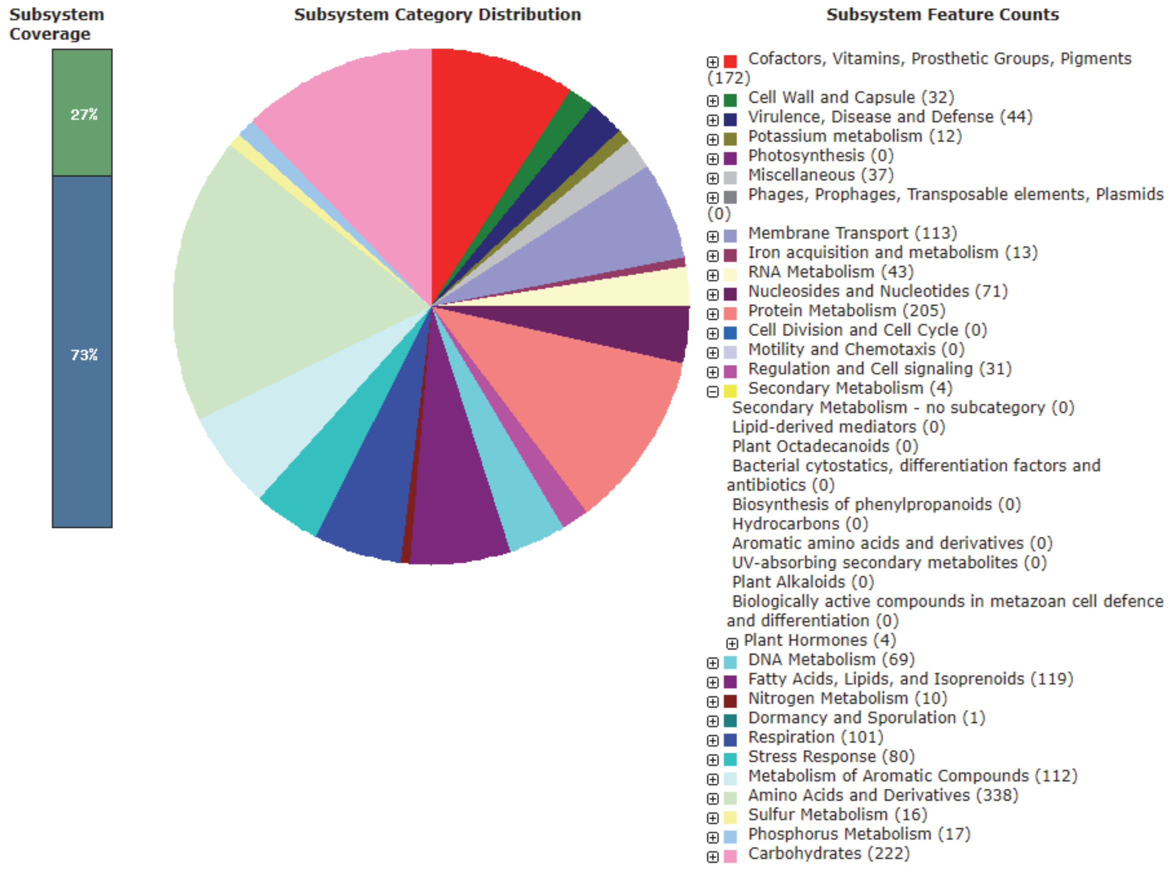

Supplementary Fig. S5. Subsystem feature of strain CYS-02^{T} revealed by RAST (Rapid Annotation using Subsystem Technology) server.

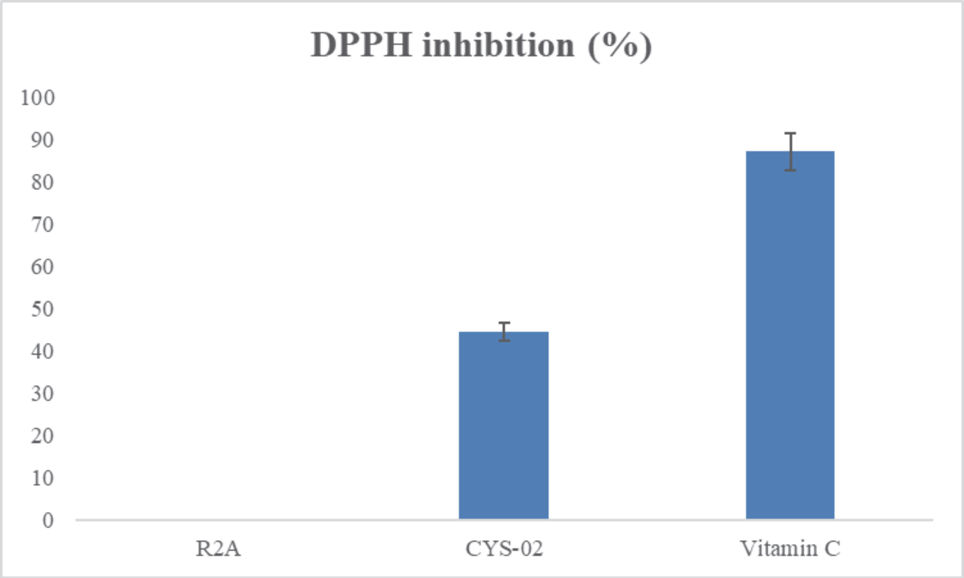

Supplementary Fig. S6. DPPH radical scavenging activities of culture supernatant of novel strain CYS-02^T. Bar represent the standard error (SE) of the triplicate samples. R2A was used as negative control and Vitamin C was used as positive control.

Supplementary Table S1. Average nucleotide identity (ANIu) and digital DNA-DNA hybridization (dDDH) between strain CYS-02^{T} and phylogenetically closest members of the genus *Variovorax*.

Strains	Accessions	II4^{T}	
		**ANIu%**	**dDDH%**
Variovorax guangxiensis DSM 27352^{T}	RXFT01000030	78.6	22.4
Variovorax paradoxus NBRC 15149^{T}	BCUT01000013	78.9	22.3
Variovorax ginsengisoli S09.D^{T}	RCZJ00000000	77.8	21.4
Variovorax soli NBRC 106424^{T}	BCUU01000176	78.4	22.1
Variovorax boronicumulans NBRC 103145^{T}	BCUS00000000	79.0	22.3
Variovorax gossypii DSM 100435^{T}	RXOE00000000	78.6	22.3

Supplementary Table S2. POCP and AAI analysis among strain CYS-02^T, and closely related strains in the genus *Variovorax* and other related species in the family *Comamonadaceae*.

**Strains**	**Accessions**	**CYS-02^T**	
		**POCP%**	**AAI%**
Variovorax guangxiensis DSM 27352^T	RXFT01000030	56.0	72.2
Variovorax paradoxus NBRC 15149^T	BCUT01000013	57.4	72.4
Variovorax ginsengisoli S09.D^T	RCZJ00000000	57.9	72.7
Variovorax soli NBRC 106424^T	BCUU01000176	57.6	72.0
Variovorax boronicumulans NBRC 103145^T	BCUS00000000	57.6	72.2
Variovorax gossypii DSM 100435^T	RXOE00000000	52.4	71.2
Caenimonas koreensis EMB320^T	WJBU01000000	58.0	74.3
Curvibacter delicatus NBRC 14919^T	BCWP01000000	56.6	75.2
Limnohabitans parvus II-B4^T	NESN01000000	54.1	72.0
Pseudorhodoferax aquiterrae KCTC 23314^T	BMYK01000000	54.7	71.7
Ramlibacter aquaticus LMG 30558^T	JADDOJ010000100	57.1	73.6
Rhodoferax saidenbachensis DSM 22694^T	CP019239	54.8	72.9
Xylophilus ampelinus CECT 7646^T	QJTC01000000	51.8	72.6

Supplementary Table S3. Subsystem feature of strain CYS-02^T and phylogenetically closet members of the genus *Variovorax* revealed by RAST (Rapid Annotation using Subsystem Technology) server.

Strains: 1, CYS-02^T; 2, *V. guangxiensis* DSM 27352^T; 3, *Variovorax paradoxus* NBRC 15149^T; 4, *Variovorax ginsengisoli* S09.D^T; 5, *Variovorax soli* NBRC 106424^T; 6, *Variovorax boronicumulans* NBRC 103145^T; 7, *Variovorax gossypii* DSM 100435^T.

**Subsystem feature counts**	**1**	**2**	**3**	**4**	**5**	**6**	**7**
Cofactors, vitamins, prosthetic groups, pigments	172	227	237	173	182	252	177
Cell wall and capsule	32	31	29	32	34	33	24
Virulence, disease and defense	44	54	50	43	48	60	38
Potassium metabolism	12	15	15	13	14	14	8
Miscellaneous	37	36	43	20	31	25	34
Phages, prophages, transposable elements, plasmids	0	3	0	1	3	0	2
Membrane transport	113	148	158	99	116	159	101
Iron acquisition and metabolism	13	14	6	7	5	23	17
RNA metabolism	43	65	54	41	55	61	51
Nucleosides and nucleotides	71	93	95	77	96	100	56
Protein metabolism	205	214	216	197	204	221	98
Motility and chemotaxis	0	16	15	1	19	15	15
Regulation and cell signaling	31	41	44	28	39	45	31
Secondary metabolism	4	7	5	4	6	5	1
DNA metabolism	69	78	89	92	83	66	63
Fatty acids, lipids, and isoprenoids	119	130	159	101	113	195	125
Nitrogen metabolism	10	14	34	8	15	13	14
Dormancy and sporulation	1	1	1	2	1	1	1
Respiration	101	122	149	115	135	141	116
Stress response	80	94	105	84	81	97	85
Metabolism of aromatic compounds	112	115	117	89	102	103	123
Amino acids and derivatives	338	438	506	322	418	525	296
Sulfur metabolism	16	18	46	7	29	22	41
Phosphorus metabolism	17	27	23	24	25	30	23
Carbohydrates	222	296	359	190	269	283	206

Supplementary Table S4. General genomic feature of strain CYS-02^T.

**Genome features**	**Value**
Genome size (bp)	4,934,485
G+C content (mol%)	67.7
No. of contigs	5
No. of subsystem	308
Total genes	4,662
CDSs (total)	4,604
tRNAs	51
rRNAs	6 (2, 2, 2)
tmRNA	1
Genome coverage	157.0×

Supplementary Table S5. The distribution of biosynthetic gene clusters (BGCs) in strain CYS-02^T.

**Region**	**Type**	**From**	**To**	**Most similar known cluster**
Region 1.1	betalactone	170,321	201,218	mycosubtilin (20%)
Region 1.2	terpene	1,366,245	1,387,981	-
Region 2.1	hserlactone	714,782	735,396	-
